# American Association of Obstetricians and Gynecologists

**Published:** 1892-11

**Authors:** L. H. Dunning

**Affiliations:** 249 N. Alabama Street, Indianapolis


### Selections.

### AMERICAN ASSOCIATION OF OBSTETRICIANS AND GYNECOLOGISTS.

[*From the Indiana Medical Journal, October.*]

The fifth annual meeting of the American Association of Obstetricians and Gynecologists was held at St. Louis, Mo., September 20th to 22d, at the Lindell hotel. There was a good attendance of members, both from the East, West, and South, and Canada was ably represented by Dr. J. F. W. Ross, of Toronto. The notable absentees were Price, Cushing, Marcy, and Krug.

The President, Dr. A. Vander Veer, presided in a most able and satisfactory manner. The moderate and judicial manner in which he engaged in the discussions was the admiration of every one present.

The programme, as published, was closely followed, and furnished ample material for scientific discussion. The papers were all good, and a few of them were of superior quality, while the discussions were animated. Altogether it was agreed that in its scientific aspect the meeting fully measured up any preceding one. It was very noticeable in the programme that the meeting was to be chiefly occupied in the consideration of surgical questions relating to obstetrics and gynecology. Such proved to be the fact of nearly all the papers and discussions.

Dr. Potter's paper on Posture brought out a lively discussion, in which a consideration of the Trendelenburg position received much attention.

Several members spoke in positive language of the advantages of the position in operations involving pelvic surgery, especially in total supra-vaginal removal of the uterus. Dr. Hoffman alone opposed its use. His ground of opposition was that it was a constrained position, that it might lead to congestion of the upper extremities and head, and to infection of the abdominal cavity by gravitation of pus in case of rupture of a pus cavity, and, furthermore, that it was entirely unnecessary.

The matter of drainage in abdominal and pelvic surgery was presented by Dr. McMurtry, and thoroughly discussed. It was the consensus of opinion that drainage should be almost universally resorted to,—first, for the purpose of drainage, and, second, as a means of indicating the presence or absence of hemorrhage. The small glass, open end, tube was preferred, though Dr. King called attention to the value of a section of the rubber tubing used in lavage. He claimed this material was firm enough to prevent collapse, and said he felt safer in its use, because he had no fear of its breaking, as he did have when he employed the glass tube. Dr. Vander Veer stated that celluloid had been proposed as a proper material, and that it commended itself to him because of its firmness, lightness, and toughness.

Several papers were presented upon the subject of Supra-vaginal Hysterectomy for Fibroid Tumors. Drs. Price and Krug's papers were not read, because of the absence of the authors, but Drs. Miller and Hall's were. The latter took strong grounds in favor of total extirpation of the uterus, and claimed that, with the patient in Trendelenburg's position, it was quite as easy of performance as the ventro-fixation operation, and had, in his hand, yielded better ultimate results.

Dr. Hoffman combated this idea, and supported his views by reference to Dr. Price's work, which showed the most rapid operations with the lowest rate of mortality of any living operator.

Dr. Reed spoke favorably of total extirpation, while Dr. McMurtry preferred ventro-fixation. Clearly, further experience is required to settle this question.

Dr. Ill, of Newark, N. J., read an elaborate and scholarly paper upon Tumors of the Abdominal Walls. His researches in this direction are so thorough and exhaustive, his paper will be found of much value.

Dr. Manton's paper on Abdominal Surgery on the Insane, and Dr. Rohé's paper on The Relations of Pelvic Diseases and Psychical Disturbances in Women, excited much interest and discussion. No brief *résumé* would do either of these papers justice. Both papers will appear in an early number of the *American Journal of Obstetrics*, and will, unquestionably, be fully discussed.

Dr. Morris's paper on Is Evolution Trying to Do Away with the Clitoris? possessed much originality, and it was discussed upon the theory of being an entirely new field of investigation. It is not so, however, as those who will carefully look up the literature of the subject will discover.

The crowning interest of the whole meeting was reached on the last afternoon, when three papers upon Ectopic Gestation were presented and elaborately discussed. Papers were read by Drs. Ross, Hall, and Rickets. Dr. Ross's paper was elaborate and exhaustive. His large experience and extensive research touching this abnormal condition entitles him to speak somewhat with authority. The salient points brought out were that extrauterine pregnancy cannot be positively diagnosticated prior to rupture; primary abdominal pregnancy may be possible, but has not been proven; interstitial pregnancy may occur; by far the vast majority of cases of ectopic gestation are tubal, and are due to preëxisting disease; rupture in tubal pregnancy occurs between the third and fourth months, and may end fatally from hemorrhage or peritonitis; or the placenta and fetus may find a new location and continue to grow until the end of the period. In the treatment of tubal pregnancy, electricity to produce death of the embryo was condemned. When a diagnosis could be made before rupture, the complete extirpation of the tubes and contents was advocated. After rupture, immediate removal is indicated.

Dr. Hoffman presented several specimens illustrating various points bearing upon the subject. One was especially interesting, it being a fetus at full term and a large-sized placenta removed from the pelvic cavity. He was unable to determine that the fetus and placenta were surrounded by a sac. The case was, prior to operation, thought to be one of normal pregnancy nearing full term, and, owing to urgent symptoms, an endeavor was made to hasten labor by introducing a catheter and rupturing the membranes. There was a discharge of amniotic fluid, but labor did not occur; finally, to save life, the abdomen was opened and the fetus and placenta found, the former in the abdominal cavity, and the placenta behind and at the side of the uterus, in the pelvic cavity. The uterus was normal, except enlarged to about the size of one at the third month of pregnancy. This was thought by many to be a case of interstitial pregnancy, and that a portion of the sac containing the amniotic fluid protruded through the uterine opening of the tube into the uterine cavity, and was ruptured by the catheter.

Dr. Ross thought that the products of gestation were surrounded by a membrane which had probably ruptured a few days before the section.

This brief report can give but a meager idea of the many excellent papers presented and the profitable discussion that followed. It is expected that all the papers will appear in a single number of the *American Journal of Obstetrics*, in November or December.

L. H. DUNNING.

249 N. Alabama Street, Indianapolis.

